# Efficacy and safety of Shexiang Baoxin Pill combined with Baduanjin in the treatment of acute myocardial infarction: A prospective randomized study

**DOI:** 10.1097/MD.0000000000044053

**Published:** 2025-08-22

**Authors:** Jianhua Fan, Zhaochen Xia, Meiqi Miao, Qiong Wu, Licheng Lu, Li Chen, Cheng Chang, Qiurong Qin, Huifen Yu, Haixiang Xu, Wen Pan

**Affiliations:** a Department of Cardiology, Kunshan Hospital of Traditional Chinese Medicine, Suzhou, Jiangsu Province, China.

**Keywords:** acute myocardial infarction, Baduanjin, cardiac function, mental health, quality of life, Shexiang Baoxin Pill

## Abstract

**Background:**

To evaluate the efficacy and safety of the Shexiang Baoxin Pill (SBP) combined with Baduanjin exercise in the treatment of patients with acute myocardial infarction (AMI).

**Methods:**

A total of 150 AMI patients who underwent percutaneous coronary intervention (PCI) were randomly assigned to 3 groups: Group A received conventional therapy combined with SBP and Baduanjin, Group B received conventional therapy with Baduanjin, and Group C received only conventional therapy. The study was conducted over 6 months, with primary outcomes measured using left ventricular ejection fraction (LVEF) for cardiac function, Hamilton Anxiety Scale (HAMA) for anxiety, Hamilton Depression Rating Scale (HAMD) for depression, and Myocardial Infarction Dimensional Assessment Scale (MIDAS) for quality of life. Safety was assessed by monitoring the incidence of adverse events.

**Results:**

Cardiac function significantly improved in all groups, with the most pronounced improvement observed in Group A (LVEF increased from 50.09% to 56.10%, *P* < .001). Group A also showed significant reductions in anxiety and depression scores (HAMA: from 18.30 to 10.07; HAMD: from 20.98 to 11.53, *P* < .05) and a substantial enhancement in quality of life (MIDAS: from 85.86 to 51.52, *P* < .05). No severe adverse events were reported.

**Conclusion:**

The integrative approach of SBP and Baduanjin with conventional therapy significantly improved cardiac function, mental health, and quality of life in patients with AMI, offering a safe and effective adjunct to conventional care.

## 1. Introduction

Acute myocardial infarction (AMI) is a critical and life-threatening condition characterized by sudden blockage of blood flow to the heart. AMI is a leading cause of morbidity and mortality worldwide, highlighting the urgent need for effective treatment strategies to enhance patient outcomes and alleviate the healthcare system’s load.^[1]^

Traditional treatments for AMI include pharmacological interventions and percutaneous coronary intervention (PCI), which have notably increased survival rates. While there remains a substantial need for complementary therapies that can enhance recovery, improve quality of life, and reduce the risk of recurrence.^[2]^

Traditional Chinese medicine (TCM) has been increasingly integrated into modern medical practices because of its holistic approach and potential benefits in managing chronic diseases, including cardiovascular conditions.^[3]^ Shexiang Baoxin Pill (SBP), a classic TCM formulation composed of 7 ingredients – artificial moschus (Shexiang), ginseng (Renshen), bovine calculus (Niuhuang), cinnamon bark (Rougui), styrax (Suxiao), toad venom (Chansu), and synthetic borneol (Bingpian) – exerts potential cardioprotective effects through multiple pathways.^[4,5]^ Its ingredients, such as artificial moschus (Shexiang) and ginseng (Renshen), have been reported to improve myocardial microcirculation by dilating coronary microvessels, thereby increasing myocardial blood perfusion and reducing myocardial ischemia. Bovine calculus (Niuhuang) and synthetic borneol (Bingpian) may exert anti-inflammatory effects by inhibiting the release of pro-inflammatory cytokines, which are crucial in mitigating myocardial injury and remodeling after AMI. Additionally, SBP can regulate lipid metabolism and stabilize atherosclerotic plaques, reducing the risk of subsequent cardiovascular events. It has been clinically used for decades in China to treat cardiovascular disorders, including coronary artery disease, heart failure, and hypertension, owing to its multitarget regulatory effects.

Baduanjin is a traditional Chinese Qigong exercise with a history of over 800 years, consisting of 8 simple and gentle movements that emphasize coordinated breathing, mental focus, and physical stretching.^[6,7]^ Its beneficial effects are thought to arise from its impact on the autonomic nervous system, particularly through the enhancement of vagal tone, which helps reduce sympathetic overactivity and lowers heart rate. Modern research has shown that regular practice of Baduanjin can improve cardiopulmonary function, reduce psychological stress, and enhance musculoskeletal flexibility.^[8,9]^ In the context of cardiovascular rehabilitation, Baduanjin has been associated with improvements in blood pressure regulation, endothelial function, and overall quality of life.^[10,11]^ These effects are particularly relevant in AMI recovery, as they contribute to both the physiological and psychological aspects of rehabilitation.

The combination of SBP and Baduanjin is hypothesized to provide a synergistic effect, where SBP’s cardioprotective properties are complemented by Baduanjin ability to improve both cardiac function and mental health. This integrative approach may lead to enhanced recovery, reduced anxiety and depression, and improved overall quality of life in patients with AMI. However, the specific combination of SBP and Baduanjin exercise for the treatment of AMI has not been extensively studied. This study aimed to evaluate the efficacy and safety of SBP combined with Baduanjin exercise in patients with AMI.

## 2. Materials and methods

### 2.1. Study design and setting

This was a prospective, single-blind randomized controlled trial to evaluate the efficacy and safety of the SBP combined with Baduanjin exercise in the treatment of patients with AMI. This experiment will follow the comprehensive trial reporting standard.^[12]^ The study was conducted from January 2021 to December 2021, enrolling a total of 182 patients from the Department of Cardiology at Kunshan Hospital of Traditional Chinese Medicine, based on the defined inclusion criteria. Of these, 32 declined to participate, and Baduanjin was performed 3 times a week for 6 months. Outcome measures were assessed both at baseline and after 6 months of intervention.

### 2.2. Ethics

This study was performed in accordance with the Code of Ethics of the World Medical Association (Declaration of Helsinki, revised in 2013) for experiments involving humans. Ethical approval was obtained from the Ethics Committee of Kunshan Hospital of Traditional Chinese Medicine (KZY2020-17), and all participants provided written informed consent.

### 2.3. Sample size and randomization

The sample size for this study was calculated using G Power software 3.1 (Heinrich Heine Universität Düsseldorf, Düsseldorf, North Rhine-Westphalia, Germany), based on the primary outcome measure: the change in left ventricular ejection fraction (LVEF). An a priori power analysis was conducted to determine the required sample size, using a 2-tailed test with a significance level (alpha) of 0.05 and a desired power of 0.80. A medium effect size (Cohen *d* = 0.5) was assumed based on a previous meta-analysis by Wei et al, which reported significant improvements in LVEF in patients with coronary heart disease treated with SBP after PCI.^[13]^ Taking into account an expected dropout rate of 10%, the final estimated sample size was set at 150 participants (50 per group) to ensure adequate statistical power.

This study adopted a single-blind randomized controlled design. While randomization was performed using a computer-generated list by an independent statistician, and allocation concealment was ensured via sealed opaque envelopes, the nature of the intervention (SBP administration and Baduanjin exercise) made it unfeasible to blind care providers and outcome participants. Thus, only the participants were blinded to the specific composition of their treatment regimen.

We acknowledge that a single-blind design may introduce potential performance and detection biases. To mitigate these limitations, we implemented the following strategies:

Blinded outcome assessors: All clinical outcome measurements, including LVEF, the Hamilton anxiety scale (HAMA), the Hamilton depression rating scale (HAMD), and the myocardial infarction dimensional assessment scale (MIDAS), were conducted by independent evaluators who were blinded to group assignments.

Standardized intervention procedures: The administration of SBP and the delivery of Baduanjin exercise followed strictly standardized protocols across all participants, reducing variability and the risk of operator-dependent bias.

Compliance monitoring: A dedicated research assistant conducted weekly follow-ups via WeChat or telephone to confirm adherence to the Baduanjin regimen, enhancing protocol fidelity.

These measures were designed to minimize potential biases associated with the single-blind design and ensure the reliability of the study findings.

### 2.4. Participants

The inclusion criteria were as follows:a confirmed AMS diagnosis according to “2023 ESC guidelines for the management of acute coronary syndrome,”^[1]^ age between 18 and 80 years, stable condition post-PCI, willingness to participate, and provision of informed consent.

The exclusion criteria included severe comorbidities likely to affect the study, allergies to any component of the SBP, inability to perform Baduanjin exercises according to the researchers’ evaluation, and current participation in other clinical trials.

The criteria for withdrawal were that the patients could not complete the exercise program or take SBP on time or voluntarily withdraw from the study.

**Figure 1.**
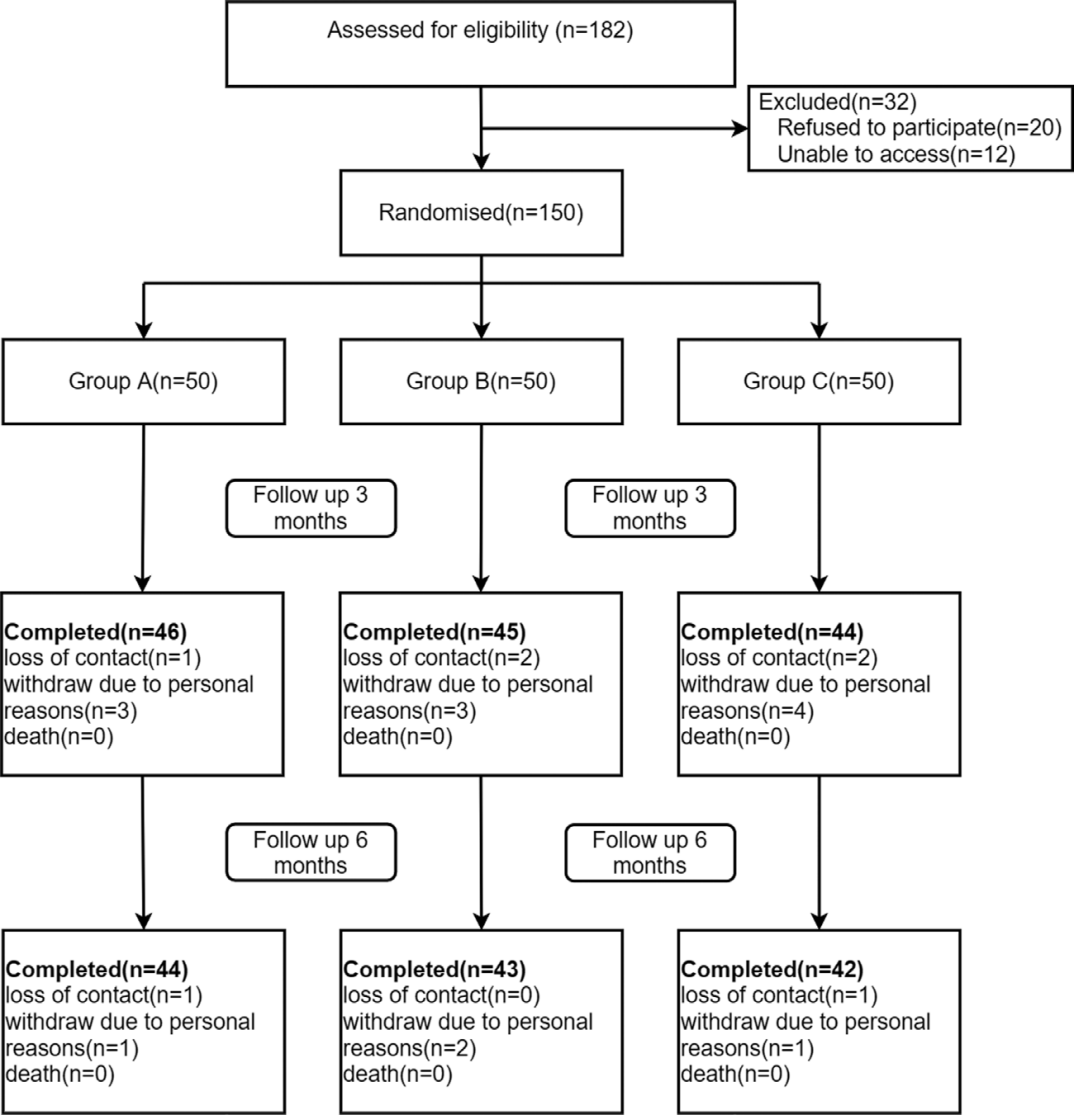
Flow diagram of the study.

A total of 150 eligible AMI participants were randomly assigned to 3 groups. Of the 150 participants,129 completed the study, resulting in an attrition rate of 14% (Fig. 1). Twenty-one patients withdrew for personal reasons (n = 14) or for loss of contact (n = 7).

### 2.5. Interventions

Group A (conventional therapy + SBP + Baduanjin):conventional drug therapy refers to standard clinical guidelines for AMI, including antiplatelet agents, beta-blockers, angiotensin-converting enzyme inhibitors, angiotensin receptor blockers, and statins. SBP (Shanghai Hutchison Pharmaceutical Company, Shanghai, China, National Medicine; batch No. 210304) was administered at a dosage of 2 pills orally 3 times daily for 6 months. Participants engaged in supervised Baduanjin sessions, 30 minutes per session, 3 times a week for 6 months. Group B (conventional therapy + Baduanjin):followed the same conventional drug therapy and Baduanjin exercise regimen as Group A. Group C (conventional therapy):received only the conventional drug therapy as described for Group A.

In accordance with the 2023 ESC guidelines for the management of acute coronary syndrome,^[1]^ conventional therapy included the following components: Dual antiplatelet therapy (DAPT): Aspirin 100 mg once daily and clopidogrel 75 mg once daily (or ticagrelor 90 mg twice daily where appropriate), initiated immediately after PCI and continued throughout the 6-month follow-up period. Beta-blockers: Metoprolol succinate 47.5 to 95 mg once daily, titrated according to blood pressure and heart rate. ACE inhibitors or ARBs: Perindopril 4 to 8 mg once daily or valsartan 80 to 160 mg once daily, adjusted based on renal function and blood pressure. Statins: Atorvastatin 20 mg once daily or rosuvastatin 10 mg once daily, initiated within 24 hours post-PCI. Other supportive medications: Nitrates, diuretics, or calcium channel blockers were prescribed as needed according to individual patient conditions.

During hospitalization, patients in group A and group B will receive regular verbal and visual demonstrations and perform Baduanjin exercises under the supervision of a cardiac rehabilitation physician. After 3 days of supervised training, participants continued to practice the same exercises independently at home 3 times a week. Each session lasted 30 minutes, which included 5 minutes of warm-up exercises followed by 25 minutes of Baduanjin practice. To ensure compliance, a research assistant communicated with participants once a week via WeChat or telephone to confirm the completion of the exercise.

The Baduanjin Qigong exercise regimen was designed according to the guidelines set by the China Sports Bureau’s Baduanjin Qigong program, comprising 8 distinct postures.^[14]^

1. Hold the sky with both hands to adjust the triple focus.
2. Bow left and right like shooting eagles.
3. To regulate the spleen and stomach must be single lifted.
4. Look back to treat damaged organs.
5. Shake your head and tail to get rid of psychological pressure.
6. Climbing feet with both hands to strengthen the kidney and waist.
7. Grow fists to increase strength.
8. Behind the 7 tiptoes to eliminate all diseases.

### 2.6. Outcomes

Outcome measures were evaluated at the beginning and after 6 months of intervention.

#### 2.6.1. Cardiac function

Cardiac function was measured by 2 fully blinded operators using echocardiography (Vivid 7, GE Healthcare, Chicago) and LVEF was measured using the Simpson method, according to the most recent guidelines from the American Society of Echocardiography.^[15]^

#### 2.6.2. Anxiety and depression levels

HAMA evaluates the severity of anxiety by assessing 14 items related to the psychological and somatic symptoms.^[16]^ The patients receive a score ranging from 0 to 4 for each item, and their overall anxiety severity was determined by the sum of these scores. A higher total score indicates a greater severity of anxiety. The scale ranges from 0 to 56, with scores below 17 indicating mild anxiety, scores between 18 and 24 indicating moderate anxiety, and scores between 25 and 30 indicating severe anxiety.

HAMD consists of 21 items, with scoring primarily based on the first 17 items.^[17]^ Eight of these items use a 5-point Likert scale, where 0 indicates the absence of symptoms and 4 indicates severe symptoms. The remaining 9 items are scored on a 0 to 2 scale. The total score from the first 17 items helps categorize the severity of depression into the following levels: normal (0–7), mild depression (8–13), moderate depression (14–18), severe depression (19–22), and very severe depression (23 or above).

#### 2.6.3. The quality of life

MIDAS comprises 35 items across 7 domains: physical activity, insecurity, emotional reaction, dependency, diet, concern over medication, and side effects. Each item is rated on a 5-point Likert scale, with 1 indicating “never” and 5 indicating “always.” The total score ranges from 0 to 140, with higher scores indicating a poorer quality of life.^[18]^

#### 2.6.4. Safety assessments

Safety was assessed throughout the study by monitoring and recording adverse events (AEs), serious adverse events (SAEs), and any other unintended medical occurrences. Safety assessments were conducted in accordance with the principles of good clinical practice and were approved by the ethics committee. The details of AEs will be recorded in the case report file and will be reported to the Ethics Committee within 24 hours.

### 2.7. Data collection

Baseline data will be collected at the start of the study, including demographic information, medical history, and baseline measures of cardiac function, anxiety, depression, and quality of life. Follow-up assessments will be conducted at 3 months and 6 months post-intervention.

### 2.8. Statistical analysis

Statistical analysis was performed using SPSS 20.0 software (IBM Corporation, Armonk). Continuous variables were presented as mean ± standard deviation and were compared using *t*-tests or the Mann–Whitney *U* test. Categorical variables were expressed as counts and percentages and were compared using the chi-square statistic or Fisher exact test for expected cell frequencies <5. Missing data were handled using last observation carried forward where appropriate. Due to the balanced baseline characteristics and limited sample size, the primary analysis was conducted using univariate comparisons. Intragroup comparisons were analyzed using paired *t*-tests, while intergroup comparisons were evaluated using 1-way analysis of variance (ANOVA). Two-tailed tests were used in all analyses and a *P*-value of <.05 was considered statistically significant. The datasets generated during and/or analyzed during the current study are available from the corresponding author on reasonable request.

## 3. Results

### 3.1. Baseline characteristics of 3 groups

A total of 150 patients diagnosed with AMI and treated with PCI were enrolled in the study and randomly assigned to 1 of 3 groups: Group A (conventional therapy + SBP + Baduanjin, n = 50), Group B (conventional therapy + Baduanjin, n = 50), and Group C (conventional therapy, n = 50). No significant differences were observed in baseline characteristics, including age, gender, medical history, body mass index, status of ST-segment elevation myocardial infarction (STEMI), stents, culprit artery and baseline measures of cardiac function, anxiety, depression, and quality of life, among the 3 groups (*P* > .05) (Table 1).

**Table 1:** The baseline characteristics of the patients (n = 150).

Characteristic	Group A (n = 50)	Group B (n = 50)	Group C (n = 50)	χ^2^/*t*	*P*
Age (yr)	54.86 ± 11.48	53.90 ± 11.70	55.62 ± 13.27	0.250	.779
Male, n (%)	31 (62%)	31 (62%)	28 (56%)	0.500	.779
Hypertension, n (%)	29 (58%)	30 (60%)	34 (68%)	1.188	.552
Diabetes mellitus, n (%)	8 (16%)	9 (18%)	10 (20%)	0.271	.873
Smoking, n (%)	15 (30%)	13 (26%)	20 (40%)	2.390	.303
BMI (kg/m^2^)	25.19 ± 1.38	25.02 ± 1.51	24.70 ± 1.70	1.141	.323
STEMI, n (%)	28 (56%)	23 (46%)	29 (58%)	1.661	.436
Culprit artery, n (%)					
LAD	29 (58%)	31 (62%)	36 (72%)	4.027	.402
LCX	4 (8%)	3 (6%)	5 (10%)		
RCA	17 (34%)	16 (32%)	9 (18%)		
Stents, n (%)					
None	2 (4%)	3 (6%)	6 (12%)	2.822	.588
One	37 (74%)	36 (72%)	32 (64%)		
Two	11 (22%)	11 (22%)	12 (24%)		
LVEF (%)	50.32 ± 5.78	48.94 ± 4.59	49.12 ± 4.70	1.102	.335
HAMA score	18.30 ± 0.95	18.26 ± 0.954	18.33 ± 1.07	0.067	.935
HAMD score	20.98 ± 1.05	20.93 ± 1.06	21.00 ± 1.14	0.050	.951
MIDAS score	85.88 ± 4.35	85.71 ± 4.37	86.21 ± 4.35	0.166	.847

BMI = body mass index, HAMA = Hamilton anxiety scale, HAMD = Hamilton depression rating scale, LAD = left anterior descending artery, LCX = left circumflex artery, LVEF = left ventricular ejection fraction, MIDAS = myocardial infarction dimensional assessment scale, RCA = right coronary artery, STEMI = ST-segment elevation myocardial infarction.

### 3.2. Cardiac function, anxiety, depression, and quality of life changes pre- and post-intervention

At 6 months, all 3 groups demonstrated improvements in cardiac function as reflected by increased LVEF. Group A exhibited the most significant improvement, with LVEF increasing from 50.09% (95% CI: 48.24 to 51.94) at baseline to 56.10% (95% CI: 54.03 to 58.17) post-intervention (*P* < .05). In comparison, LVEF in Group B rose from 48.84% (95% CI: 47.35 to 50.33) to 52.74% (95% CI: 51.14 to 54.34), while Group C showed a smaller increase from 48.90% (95% CI: 47.40 to 50.40) to 50.86% (95% CI: 49.30 to 52.42). Between-group comparisons indicated that the improvement in Group A was significantly greater than in Groups B and C (*P* < .05).

Anxiety and depression levels, assessed using the HAMA and HAMD scales, respectively, showed significant reductions in all groups. Group A’s HAMA score decreased from 18.30 (95% CI: 18.01 to 18.59) to 10.07 (95% CI: 9.91 to 10.23), while the HAMD score dropped from 20.98 (95% CI: 20.66 to 21.30) to 11.53 (95% CI: 11.36 to 11.70) (*P* < .05). Group B demonstrated moderate improvements, and Group C showed the least favorable changes, with statistically significant differences between groups.

Quality of life, measured by MIDAS, also improved most markedly in Group A, with scores decreasing from 85.86 (95% CI: 84.53 to 87.19) to 51.52 (95% CI: 50.72 to 52.32). Group B improved from 85.57 (95% CI: 84.22 to 86.92) to 59.90 (95% CI: 58.96 to 60.84), while Group C changed from 86.23 (95% CI: 84.88 to 87.58) to 68.98 (95% CI: 67.90 to 70.06). Again, post-intervention scores in Group A were significantly better than those in Groups B and C (*P* < .05). Detailed values and confidence intervals are presented in Table 2.

**Table 2:** Changes in LVEF, HAMA, HAMD, and MIDAS scores at baseline and 6 months post-intervention (mean and 95% confidence intervals).

Group	Case	Time	LVEF (%) (95% CI)	HAMA (95% CI)	HAMD (95% CI)	MIDAS (95% CI)
A	44	Baseline	50.09 (48.24–51.94)	18.30 (18.01–18.59)	20.98 (20.66–21.30)	85.86 (84.53–87.19)
A	44	6 mo	56.10 (54.03–58.17)*	10.07 (9.91–10.23)*	11.53 (11.36–11.70)*	51.52 (50.72–52.32)*
B	43	Baseline	48.84 (47.35–50.33)	18.24 (17.95–18.53)	20.90 (20.58–21.22)	85.57 (84.22–86.92)
B	43	6 mo	52.74 (51.14–54.34)*,†	12.77 (12.57–12.97)*,†	14.63 (14.40–14.86)*,†	59.90 (58.96–60.84)*,†
C	42	Baseline	48.90 (47.40–50.40)	18.34 (18.03–18.65)	21.01 (20.67–21.35)	86.23 (84.88–87.58)
C	42	6 mo	50.86 (49.30–52.42)†,‡	14.68 (14.43–14.93)*,†,‡	16.81 (16.54–17.08)*,†,‡	68.98 (67.90–70.06)*,†,‡

CI = confidence interval, HAMA = Hamilton anxiety scale, HAMD = Hamilton depression rating scale, LVEF = left ventricular ejection fraction, MIDAS = myocardial infarction dimensional assessment scale.

* *P* < .05 vs before intervention in the same group.

† *P* < .05 vs group A 6 months.

‡ *P* < .05 vs group B 6 months.

### 3.3. Safety

No AEs or SAEs related to Baduanjin or SBP occurred in this trial.

## 4. Discussion

This study aimed to assess the efficacy and safety of integrating SBP with Baduanjin exercises in the management of patients with AMI. The findings indicated that this combined intervention not only significantly enhanced cardiac function but also positively influenced the mental health and overall quality of life of the participants. Notably, patients in the combined therapy group (Group A) experienced a substantial increase in LVEF, from 50.09% at baseline to 56.10% post-treatment, a change that was markedly superior to that of the other groups, underscoring the superior cardiac recovery associated with the integrated approach.

In addition to cardiac benefits, the combined therapy significantly alleviated levels of anxiety and depression as evaluated by HAMA and HAMD. Group A participants demonstrated a significant reduction in HAMA scores from 18.30 at baseline to 10.07 after 6 months, and HAMD scores from 20.98 to 11.53 over the same period. These improvements were notably greater than those in groups receiving only conventional therapy or a combination of conventional therapy with Baduanjin. The mental health benefits are particularly significant, suggesting that the combined therapy addresses both the physiological and psychological facets of AMI recovery. Furthermore, the quality of life, as measured by the MIDAS, showed a substantial improvement in the combined therapy group, with scores decreasing from 85.86 to 51.52. This enhancement underscores the comprehensive benefits of the SBP and Baduanjin integration and offers a multifaceted strategy for patient recovery. Future studies should incorporate qualitative interviews or focus groups to explore patient experiences with the combined therapy, as well as additional quality of life measures such as the SF-36 or EQ-5D to comprehensively evaluate physical, psychological, and social outcome.

Importantly, no SAEs were reported across any treatment groups throughout the study, indicating that the combined use of SBP and Baduanjin was both efficacious and safe for AMI recovery. The lack of significant adverse effects supports the potential of this combined therapy as a viable and safe complement to the standard AMI treatment regimens. Although no AEs or SAEs related to SBP or Baduanjin exercise were reported during the 6-month follow-up period, we acknowledge the limitations of the current safety assessment. Due to the relatively short follow-up duration and modest sample size, the study may not have been adequately powered to detect rare or delayed-onset adverse effects. While our findings support the safety of SBP and Baduanjin in the short term, it is important to recognize that the 6-month follow-up period and sample size of 150 patients may not capture infrequent or delayed AEs. This limits our ability to draw definitive conclusions about the long-term safety of the intervention.

The study’s findings corroborate and extend the existing body of literature on the cardiovascular benefits of SBP and Baduanjin, especially for AMI patients. Previous studies have extensively documented the cardioprotective effects of SBP, primarily attributing its benefits to improved myocardial microcirculation, reduced myocardial oxygen consumption, and its anti-inflammatory properties.^[19,20]^ These effects have been particularly noted in patients with chronic heart failure and coronary artery disease, in whom SBP has been shown to enhance cardiac function and alleviate symptoms.^[21,22]^ Similarly, Baduanjin, consisting of gentle movements and breathing exercises, is designed to enhance energy flow, reduce stress, and promote overall well-being.^[23]^ Research has demonstrated that regular Baduanjin practice can improve cardiovascular function, increase exercise capacity, and enhance quality of life, especially in patients with chronic heart conditions.^[24,25]^ The synergistic effects of SBP and Baduanjin may be attributed to multiple mechanisms. SBP’s ingredients have been shown to exert endothelial protection, reduce myocardial inflammation, attenuate cardiac remodeling, and modulate lipid metabolism.^[19,26]^ Concurrently, Baduanjin complements these effects through autonomic nervous system regulation^[27]^: its slow, rhythmic movements and diaphragmatic breathing enhance vagal tone, reducing sympathetic overactivity to lower resting heart rate and myocardial oxygen demand. This dual intervention of SBP’s direct pharmacological actions and Baduanjin mind-body regulation likely amplifies the improvements in LVEF and quality of life observed in Group A, addressing both the structural and functional pathways of AMI recovery.

In addition to the physiological improvements in cardiac function, the combined intervention also demonstrated notable benefits in reducing anxiety and depression and improving overall quality of life. These effects may be partially explained by the neuroendocrine and psychosocial impacts of SBP and Baduanjin. SBP may also exert central nervous system effects through components such as ginseng and synthetic borneol, which have been shown to cross the blood-brain barrier and modulate neurotransmitter systems involved in mood regulation. Baduanjin, as a mind-body exercise, contributes to lower levels of cortisol and other stress-related hormones. This autonomic modulation has been associated with improvements in mood and anxiety symptoms. Furthermore, the meditative and rhythmic components of Baduanjin may foster emotional regulation and mindfulness, contributing to psychological resilience during postinfarction recovery. Moreover, the structured rehabilitation environment, weekly communication with healthcare staff, and perceived self-efficacy in performing Baduanjin may create a supportive psychosocial context that reinforces behavioral engagement and emotional well-being. These multimodal mechanisms likely underlie the significant improvements observed in HAMA, HAMD, and MIDAS scores in the combined intervention group.

However, prior to this study, the specific combination of SBP and Baduanjin in the context of AMI treatment had not been extensively studied. Our research fills this gap by showing that the combined use of SBP and Baduanjin leads to significantly greater improvements in cardiac function, anxiety, depression, and quality of life than conventional therapy alone or in combination with Baduanjin. This synergistic effect suggests that the mechanisms of SBP and Baduanjin may complement each other, resulting in enhanced therapeutic outcomes. Although this study demonstrated significant clinical improvements, the underlying biological mechanisms remain to be clarified. Future studies should incorporate biomarker analysis – such as inflammatory cytokines, oxidative stress indicators, and neuroendocrine markers – as well as gene expression profiling, to elucidate the molecular pathways involved in the combined therapeutic effects of SBP and Baduanjin.

Despite the promising results of this study, several limitations must be acknowledged. First, this was a single-center trial conducted at Kunshan Hospital of Traditional Chinese Medicine, which may limit the generalizability of the findings to broader or more diverse patient populations. Multicenter studies involving different healthcare systems, ethnic groups, and care models are needed to validate and extend our conclusions. Second, although the sample size of 150 patients was statistically adequate to detect significant intragroup changes, it may not have been powered sufficiently to detect smaller intergroup differences or to support subgroup analyses (e.g., by age, gender, or infarct type). The relatively modest cohort size also limits the external validity and scalability of our findings. Third, the 6-month follow-up period is relatively short in the context of cardiovascular recovery, particularly with respect to assessing the long-term sustainability of improvements in cardiac function and psychological well-being, as well as the potential emergence of delayed AEs. A longer follow-up period (e.g., 12–24 months) is necessary to assess the durability and safety of these interventions. Fourth, adherence to Baduanjin exercise was self-reported and monitored via weekly telephone or WeChat check-ins, which may introduce recall and social desirability biases. The use of wearable devices or digital activity trackers in future studies could allow for more accurate and objective assessment of patient engagement. Fifth, the study employed a single-blind design. Due to the nature of the intervention, it was not feasible to blind care providers or patients entirely, raising the possibility of performance or detection bias. While outcome assessors were blinded, the lack of double-blinding remains a methodological limitation. Sixth, our analysis did not incorporate multivariate adjustment or interaction modeling, which may have provided greater insight into the independent and combined effects of SBP and Baduanjin. Future research should utilize more advanced statistical approaches to isolate these effects and identify potential moderators.

To strengthen the clinical applicability of our findings, future research should focus on large-scale, multicenter randomized controlled trials that include diverse patient populations across different healthcare settings. These studies should extend the follow-up duration to at least 12 months to evaluate the long-term sustainability of the observed benefits and detect delayed AEs. Objective adherence monitoring using wearable activity trackers and mobile health technologies should be incorporated to enhance data reliability. In addition, future analyses should employ multivariate and interaction models to better elucidate the independent and synergistic effects of SBP and Baduanjin. Where feasible, double-blind designs using placebo and sham-exercise controls should be considered to minimize bias. Comparative studies with other non-pharmacological interventions may further clarify the role of this integrative approach within contemporary cardiac rehabilitation frameworks.

## 5. Conclusion

This study provides promising evidence supporting the integrated use of SBP and Baduanjin exercise as a safe and effective adjunctive therapy for patients with AMI. The observed improvements in cardiac function, mental health, and quality of life suggest a synergistic benefit of this combined approach. However, these findings should be interpreted with caution due to the study’s single-center design and limited follow-up. Further large-scale, multicenter randomized trials with extended follow-up periods are needed to confirm the long-term efficacy and safety of this integrative intervention and to validate its applicability across broader and more diverse patient populations.

## Acknowledgments

The authors express heartfelt thanks to all the patients who participated in this study. The authors also thank the staffs at the cardiology department for their enthusiasm for this study.

## Author contributions

**Conceptualization:** Wen Pan.

**Data curation:** Jianhua Fan, Zhaochen Xia.

**Formal analysis:** Zhaochen Xia, Qiong Wu, Huifen Yu.

**Investigation:** Licheng Lu, Li Chen, Qiurong Qin.

**Methodology:** Cheng Chang.

**Project administration:** Wen Pan.

**Validation:** Haixiang Xu.

**Visualization:** Qiong Wu, Huifen Yu.

**Writing – original draft:** Jianhua Fan, Meiqi Miao.

**Writing – review & editing:** Haixiang Xu, Wen Pan.
